# Hospital Abuse

**Published:** 1906-12-15

**Authors:** Henry Cartmel

**Affiliations:** Cleveleys


### Editor's Letter-Box.

[Our Correspondents are reminded that prolixity is a great bar to publication, and that brevity of style and conciseness of statement greatly facilitate early insertion.]

### HOSPITAL ABUSE.

Sir,—You are placing the medical profession, and especially the general practitioner, under a heavy debt of gratitude for the able and consistent manner in which you have exposed the abuses attending the management of our different charitable institutions, and more particularly our large hospitals and other medical associations. You ought to be supported, and I, for one, humbly wish to offer you my sincere thanks and congratulations. So long ago as June 1873, when provident dispensaries were being started in Manchester, I wrote to the *Manchester Courier* protesting against the need of them. I then argued that, "if the kind of patients attending our hospitals were divided into three classes, two might, and ought to, be excluded," and thus "do away with any necessity of starting other medical associations, by which the interests of the general practitioner would be greatly damaged."

The "classes," or divisions, I then made were: "First, those who were in a position to pay a private medical practitioner. Second, those who, unfortunately, had not the means to procure necessary nourishment during their illness, and therefore proper cases for parochial care. And, third, those who, through accident or disease, were, for a time, thrown out of employment, and, though both able and willing at other times to pay their doctor, would not then be able to do so without incurring a debt that would be a terrible hindrance to their future prospects." No doubt the last class is the one principally intended to be helped by those who subscribe to our hospitals; and the others constitute a very great abuse. But the apathy of a great number of our medical brethren, as well as of the subscribers, has been such that the abuse has been allowed to go on, with nothing more than a little grumbling, until at last there appears to be some hope of a kind of a revolution, which I trust will meet with tangible help and success, so that the general medical practitioner and the public may be placed in their right positions.

All thanks to you, Sir. Yours, etc.,

Henry Cartmel, M.R.C.S.

Cleveleys, December 3, 1906.

